# Exposure to renin-angiotensin system inhibitors before kidney transplantation is associated with a decreased risk of delayed graft function

**DOI:** 10.3389/fimmu.2024.1447638

**Published:** 2024-11-22

**Authors:** Gan Zhen Liang, Marc Dorais, Suzon Collette, Lynne Sénécal, Majda Belkaid, Julie Turgeon, Héloïse Cardinal

**Affiliations:** ^1^ Department of Surgery, Université de Montréal, Montreal, QC, Canada; ^2^ Research center, Centre Hospitalier de l’Université de Montréal (CRCHUM), Montreal, QC, Canada; ^3^ Department of Medicine, Hôpital Maisonneuve-Rosemont, Montréal, QC, Canada; ^4^ StatSciences Inc, Notre-Dame de l’Ile-Perrot, QC, Canada; ^5^ Department of Medicine, Université de Montréal, Montreal, QC, Canada; ^6^ University of Alberta, Canadian Donation and Transplantation Research Program, Edmonton, AB, Canada

**Keywords:** renin-angiotensin-aldosterone system blockers, delayed graft function, kidney transplantation, acute kidney injury, chronic kidney disease

## Abstract

**Introduction:**

Animal models suggest a protective role of angiotensin-converting enzyme inhibitors (ACEi) and angiotensin-II receptor blockers (ARBs) in reducing renal and cardiac ischemia-reperfusion injury. Our aim was to determine the association between pre-transplant ACEi/ARBs use and the occurrence of delayed graft function (DGF) in patients who received a kidney transplantation from a deceased donor.

**Methods:**

Consecutive recipients between 2008 and 2021 in 2 Canadian university-affiliated centers were included in this retrospective cohort study. The main outcome was the occurrence of DGF and the exposure was use of ACEi or ARBs at the time of admission for transplantation. Mixed models were fit.

**Results:**

A total of 897 patients were included, of which 160 (18%) experienced DGF. At admission, 337 (38%) patients were exposed to ACEi/ARBs. In the multivariable analysis, pre-transplant ACEi/ARBs use was associated with a reduced risk of DGF (odds ratio: 0.60, 95% confidence interval: 0.40, 0.92). Other factors associated with DGF were recipient obesity, donor type, ethnicity, age, hypertension, and total ischemia time.

**Discussion:**

Pre-transplant use of ACEi/ARBs is associated with a lower risk of DGF in early postoperative period, which may be due to a protective effect of these agents on renal ischemia-reperfusion injury.

## Introduction

The advantages of using angiotensin-converting enzyme inhibitors (ACEi) and angiotensin-II receptor blockers (ARBs) in patients with chronic kidney disease (CKD) are well documented. These agents inhibit the renin-angiotensin-aldosterone system (RAAS) and slow down the progression of CKD by limiting functional and structural changes (1). However, they can aggravate acute kidney injury (AKI) during hemodynamic instability, hypotension or volume depletion (2). Hence, their discontinuation in the setting of AKI is recommended (3).

In the context of kidney transplantation, postoperative AKI, or delayed graft function (DGF), is most often caused by ischemia-reperfusion injury (IRI) that occurs in association with donor hypotension, organ recovery, cold storage and anastomosis. DGF is associated with prolonged hospitalization, increased risk of acute rejection, lower long-term graft function, and decreased allograft survival (4). Due to fear of hyperkalemia, hemodynamic instability and volume depletion in the polyureic phase of acute tubular necrosis, the current practice in our centers is to discontinue ACEi and ARBs after transplant, at least for the first 2 weeks.

Animal models suggest that blocking the RAAS can be protective in the context of cardiac (5), as well as renal IRI (6–10). Pre-treatment with ARBs or ACEi before renal IRI in male Wistar-Albino rats led to a decreased severity of functional injury (lower serum urea and creatinine), lower asymmetric dimethylarginine (ADMA) levels, increased superoxide dismutase and glutathione peroxidase activities, suggesting that these agents are protective in renal IRI through an antioxidant activity (10). In another study, rats that were pre-treated by ARBs prior to renal IRI showed lower creatinine levels, lower circulating interleukin-6 (IL-6) and tumor necrosis factor alpha (TNF-α) levels, as well as increased renal expression of anti-apoptotic Bcl-2 combined with lower expression of pro-apoptotic Bax and caspase-3, suggesting that ARBs could also protect the kidney from IRI though anti-inflammatory and anti-apoptotic activities (9). Blocking the RAAS in deceased kidney donors 10 minutes before the time of organ recovery has also been associated with a lower risk of DGF in kidney transplant recipients (11). Hence, recipient use of long-acting ACEi and ARBs in the pre-transplant context may be beneficial to prevent DGF, even if they are discontinued post-transplant. Here, our aim was to determine the association between ACEi and ARBs use at the time of admission for kidney transplantation and the occurrence of delayed graft function (DGF) in the postoperative period.

## Methods

### Patients and setting

We performed a retrospective cohort study in 2 Canadian, university-affiliated adult kidney transplant centers (Centre hospitalier de l’Université de Montréal and Hôpital Maisonneuve-Rosemont). Consecutive patients who received a kidney transplant between July 1^st^, 2008 and July 1^st^, 2021 and accepted to participate in the clinical and biological database of the University of Montreal Renal Transplant Biobank were screened for inclusion. Recipients of living donors and of combined solid organ transplants were excluded. This project was approved by the local ethics review board of the Centre hospitalier de l’Université de Montréal (MP-02.2023-10828).

### Measurements

The primary outcome, DGF, was defined as the need for dialysis in the first postoperative week, which is the definition most commonly used (12). We also examined a second definition for DGF which was less strict and included slow graft function. In this second definition, DGF was defined as having one of the following 3 criteria: the need for dialysis in the first week post transplant, the failure of serum creatinine to decrease by 10% or more in the first 3 days post transplant, or serum creatinine over 250 umol/l on post-transplant day 5 in the presence of scintigraphic evidence of acute tubular necrosis (13). We have found this definition to be associated with lower graft function 1 year post transplant (13), which in turn is strongly associated with kidney graft survival (14).

Our main independent variable was the use of ACEi or ARBs at the time of admission for transplantation. Recipient-related covariables were age at transplant, sex, race, obesity, diabetes, cytomegalovirus (CMV) serology, smoking status, cause of CKD, previous transplantation, current panel reactive antibodies. Donor-related covariables were donor type (neurologically deceased versus after cardiocirculatory arrest), age, sex, hypertension, diabetes, human leukocyte antigens (HLA) mismatch, smoking status, terminal serum creatinine. Procedure-related covariables were use of hypothermic perfusion pump, center, total ischemia time and induction agent. Basiliximab is the standard induction therapy in both centers while thymoglobulin is given if the treating physician perceives the recipient to be at higher risk or rejection and/or DGF.

### Statistical analyses

Continuous variables are reported as medians and interquartile ranges according to their non-normal distribution. Categorical variables are presented as proportions. We performed chi-square tests to assess differences in proportions for categorical variables and Wilcoxon rank sum tests for differences in continuous variables. All regression analyses were performed using mixed models to take into account the correlated nature of the data, as 258 recipients received kidneys originating from the same donor (129 pairs). We fit univariable mixed models to evaluate the associations between the main exposure (ACEi or ARBs use at time of admission), the covariables listed above, and the dependent variable (DGF). Then, we fit a multivariable mixed model to evaluate the independent association between the use of ACEi or ARBs at admission for transplant and the occurrence of DGF. All variables that were associated with the outcome (DGF) with a p-value <0.15 on univariable analyses (**Supplementary Table 1**) as well as those associated with the exposure (ACEi/ARBs at admission) with a p-value <0.15 (**Table 1**) were included in the multivariable model. The same procedures were repeated for the extended definition of DGF.

**Table 1:** Recipient and donor characteristics amongst study participants stratified by the use of ACEi/ARBs at the time of admission for transplantation (n=897 unless specifies otherwise*).

Characteristics	ACEi/ARBs users (n=337)	ACEi/ARBs nonusers (n=560)	*p*-value
Recipient			
Median age at transplant in years (Interquartile range (IQR))	52 (42-62)	55 (46-63)	0.04
Male sex, n (%)	215 (64)	352 (63)	0.77
African American race, n (%)	33 (10)	55 (10)	0.69
Obesity, n (%)	75 (22)	146 (26)	0.20
Cause of chronic kidney disease, n (%)			0.25
Glomerular diseases	126 (32)	181 (37)	
Diabetes	60 (18)	94 (17)	
Hypertension/vascular	38 (11)	51 (9)	
Polycystic kidney diseases	44 (13)	95 (17)	
Autoimmune diseases	12 (4)	27 (5)	
Positive CMV serology, n (%)	175 (52)	289 (52)	0.93
Pretransplant diabetes, n (%)	98 (29)	148 (26)	0.39
Coronary artery disease at transplantation, n (%)	60 (18)	99 (18)	0.96
Active smoking at transplantation, n (%)	60 (18)	67 (12)	0.02
Past history of smoking, n (%)	126 (38)	242 (43)	0.08
Statin use at transplantation, n (%)	185 (55)	294 (53)	0.49
Flow class 1 pre-transplant panel reactive antibodies˃0%, n (%)	117 (35)	186 (33)	0.49
Flow class 2 pre-transplant panel reactive antibodies˃0%, n (%)	73 (22)	133 (24)	0.49
First transplantation, n (%)	298 (88)	497 (89)	0.88
HLA mismatches, n (%)			0.26
3-4	169 (50)	294 (53)	
5-6	104 (31)	183 (33)	
Previous transfusion, n (%)	137 (38)	231 (41)	0.26
Previous pregnancy, n (%)*	94 (28)	152 (27)	0.91
Induction with thymoglobulin, n (%)	97 (29)	156 (28)	0.77
Donor			
Donor after cardiocirculatory arrest, n (%)*	54 (16)	105 (19)	0.31
Mean age in years, (SD)	48 (16)	49 (16)	0.15
Male sex, n (%)	190 (56)	320 (57)	0.83
Median height in meters (IQR)	1.70 (1.63-1.78)	1.70 (0.1.61-1.77)	0.38
Positive CMV serology, n (%)	130 (38)	210 (38)	0.99
Diabetes, n (%)*	28 (8)	43 (8)	0.36
Hypertension, n (%)*	94 (28)	141 (25)	0.61
Tobacco history, n (%)*	185 (55)	325 (58)	0.39
Donor vascular disease, n (%)*	38 (11)	53 (10)	0.53
Median terminal serum creatinine in µmol/L (IQR)*	63 (48-80)	60 (48-75)	0.07
Procedure			
Use of hypothermic perfusion pump, n (%)	113 (78)	211 (78)	0.89
Center 1, n (%)	173 (51)	291 (52)	0.86
Median total ischemic time in hours (IQR)	11 (8.6-14.2)	11 (8-14.4)	0.54

*Missing data. Pregnancy n=3, donor diabetes n=38, donor hypertension n=33, donor smoking n=36, donor peripheral vascular disease n=53, donor creatinine n=43, donor type (neurologically deceased vs after cardiocirculatory arrest n=3).

Afterwards, to better account for confounding by donor-related variables, we performed a subgroup analysis of recipient pairs who received kidneys from the same donor but where only one of the recipients was exposed to ACEi/ARB pre-transplant. In these discordant pairs, we fit a univariable mixed model with DGF as the dependent variable and ACEi/ARB exposure as the independent variable.

We then explored whether the associations were due to a class effect by fitting univariable mixed models for exposure to ACEi alone and ARB alone pre-transplant. Last, we explored whether there was a dose-response relationship in exposure to ACEi, ARBs and DGF. To achieve this, we converted specific ACEi to equivalent daily doses of ramipril 2.5 mg per day (which was then considered 1 unit for ACEi exposure), which was then adjusted for renal clearance (15). In a similar fashion, we converted all ARBs to equivalent daily doses of irbesartan 150 mg per day (which was then considered 1 unit for ARBs exposure) (16). We dichotomized the equivalent ACEi and ARB doses according to whether they were equal to or below (low dose) or above (high dose) the median of their respective distributions. We then fit 2 univariable mixed models where the reference categories were ‘no exposure to ACEi nor ARBs pre-transplant’ and the independent variables were low-dose and high-dose ACEi use in one model. In the other model, the independent variables were low-dose and high dose ARB use. Analyses were performed using SAS v9.4 (Cary, NC).

## Results

**Figure 1:**
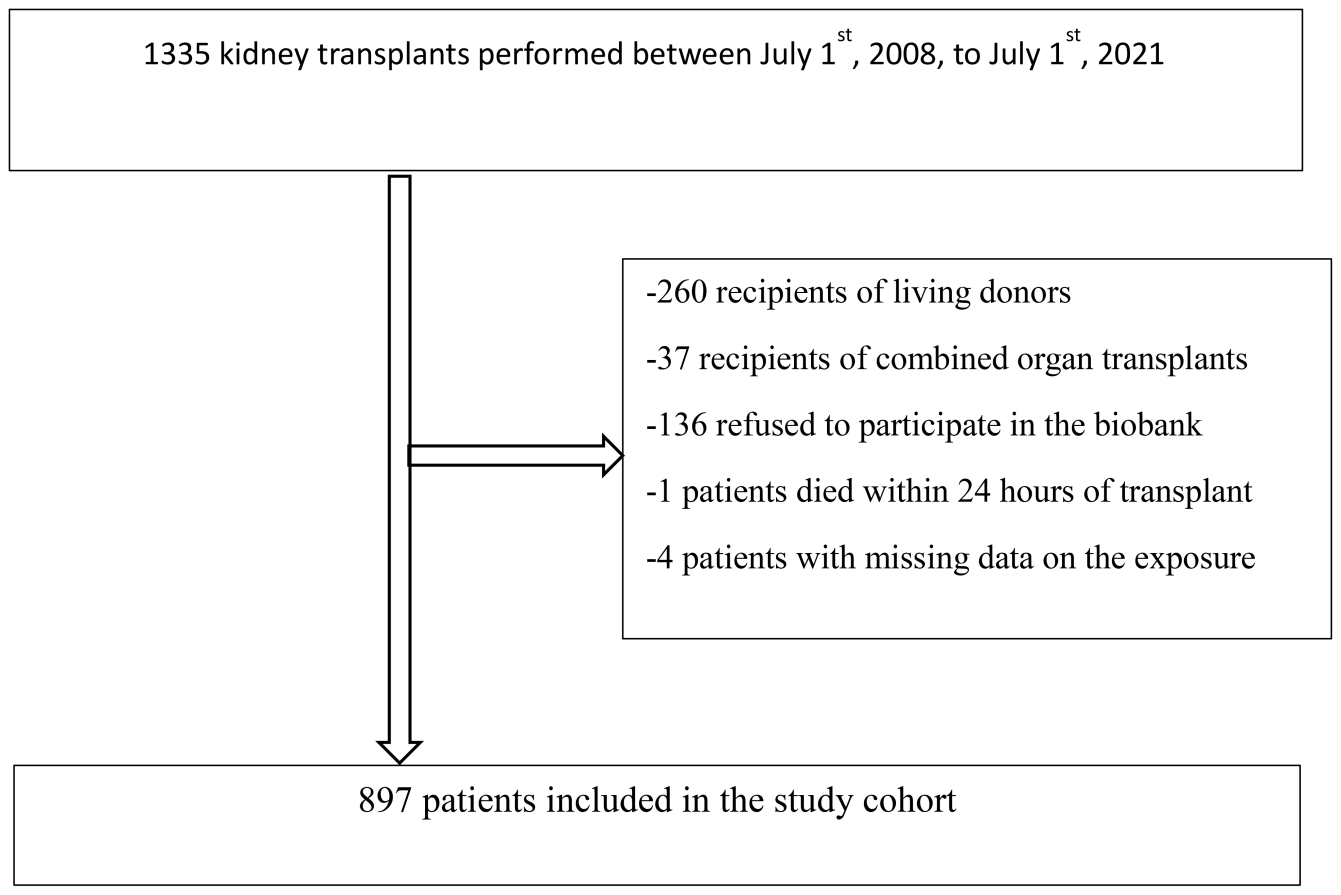
Patient flow chart.

**Figure 2:**
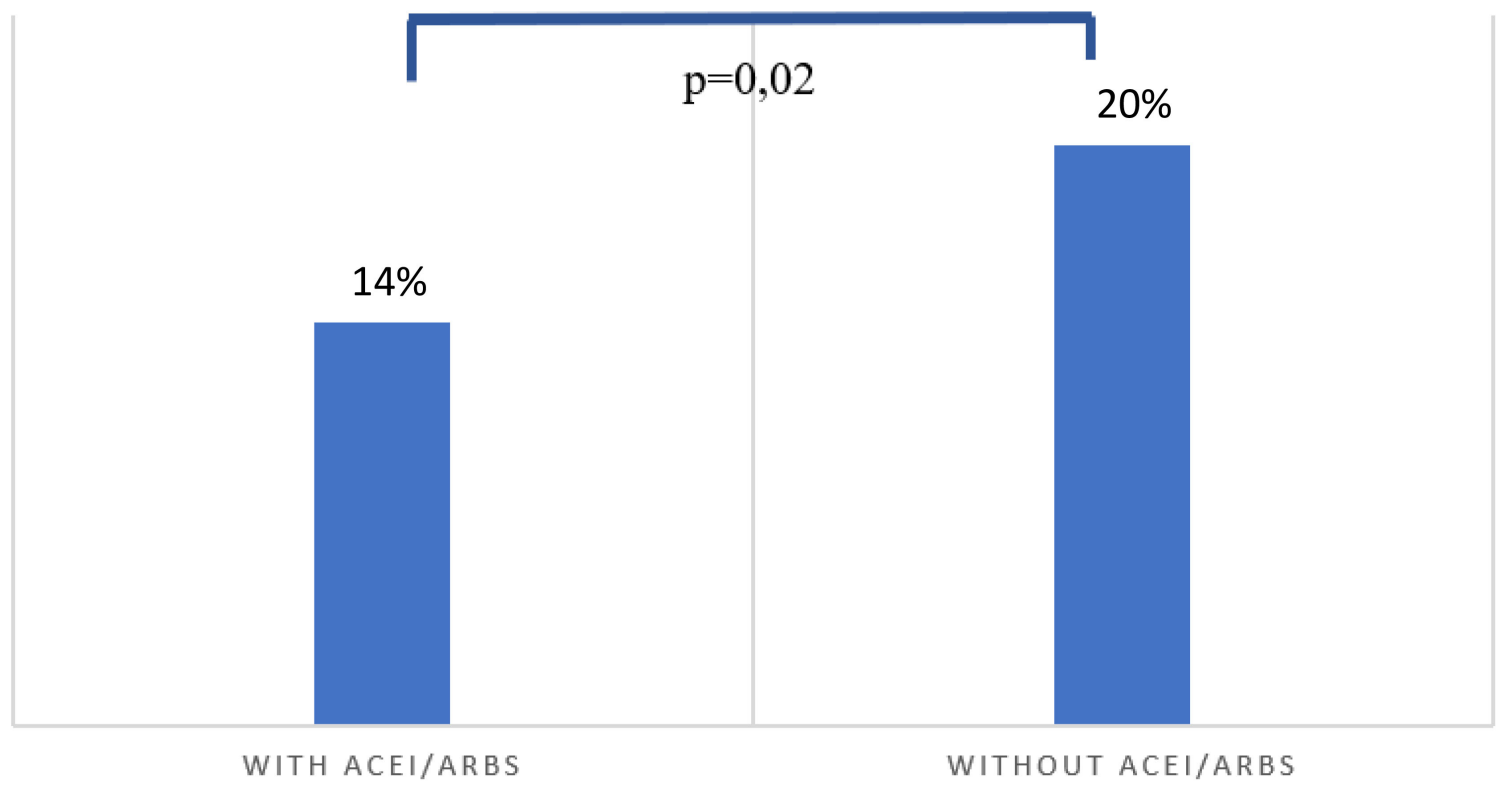
Occurrence of delayed graft function according to pre-operative exposure to renin-angiotensin system blockers [angiotensin-converting enzyme inhibitors (ACEi) or angiotensin-II receptor blockers (ARBs)]. Amongst ACEi/ARBs users, 49 (14%) recipients experienced delayed graft function whereas in nonusers, 114 (20%) patients experienced DGF (p-value=0.02).

After the exclusion criteria were applied, a total of 897 patients were included in the study cohort (**Figure 1**). Amongst the latter, 160 (18%) experienced DGF. **Table 1** presents recipient, donor and procedure-related characteristics in kidney transplant recipients stratified by the use of ACEi/ARBs at the time of transplantation. At the time of admission, 337 (38%) patients were exposed to ACEi/ARBs while 560 (62%) patients were not exposed. Most characteristics were similar between ACEi/ARBs users and nonusers, but users were younger at transplantation (52 *vs* 55 years, p-value 0.04) and were more likely to be smokers (18% *vs* 12%, p=0.02). Amongst ACEi/ARBs users, 47 (14%) recipients experienced DGF whereas in nonusers, 113 (20%) patients experienced DGF (p-value=0.02) (**Figure 2**). Median length of hospital stay was similar in patients exposed to ACEi/ARBs (13 days, interquartile range (IQR) 10-20 days) and those who were not exposed (14 days, IQR 10-20 days) (p=0.75). The incidence of rejection in the first month after transplantation was low and similar in recipients exposed and non-exposed to ACEi/ARBs (7% versus 6% respectively, p=0.34)

In the multivariable analysis, pre-transplant ACEi/ARBs use was associated with a reduced risk of DGF (odds ratio (OR): 0.60, 95% confidence interval (CI): 0.40, 0.92) (**Table 2**). Recipient African American ethnicity (OR: 2.22, 95% CI 1.18, 4.18), recipient obesity (OR 2.76, 95% CI 1.85, 4.11), donor after cardio-circulatory arrest (OR 3.50, 95% CI 2.09, 5.86), donor age (OR 1.16 for every 10 years older, 95% CI 1.00, 1.34), donor hypertension (OR 1.55, 95% CI 1.00, 2.37), and total ischemia time (OR 1.04 for every additional hour, 95% CI 1.00, 1.09) were also associated with an increased risk of DGF. We present the multivariable analysis without use of thymoglobulin for induction, as the latter is often used when there is already a suspicion of DGF at our centers, but results were similar when thymoglobulin was included in the model. Results of all univariable analyses can be found in **Supplementary Table 1**.

**Table 2:** Associations between recipient, donor, and procedure characteristics and DGF in final multivariable analyses (n=897).

Characteristics	Multivariable odds ratio (95% CI*)	*p*-value
Recipient ACEi/ARBs use at transplantation	0.60 (0.40, 0.92)	0.02
Recipient age at transplant (*per 10 years higher*)	0.85 (0.71, 1.01)	0.07
Recipient African American ethnicity *(vs all other ethnicities)*	2.22 (1.18, 4.18)	0.01
Recipient obesity	2.76 (1.85, 4.11)	<0.01
Recipient diabetes	0.73 (0.40, 1.33)	0.31
Recipient positive CMV serology	1.48 (0.98, 2.23)	0.06
Recipient active smoking at transplantation *(vs never smoked)*	0.91 (0.49, 1.69)	0.77
Recipient past history of smoking *(vs never smoked)*	1.15 (0.75, 1.76)	0.53
Cause of CKD* glomerular disease (vs other/unknown)	1.47 (0.82, 2.63)	0.20
Cause of CKD hypertension/vascular (vs other/unknown)	1.64 (0.74, 3.62)	0.22
Cause of CKD polycystic kidney disease (vs other/unknown)	0.89 (0.41, 1.91)	0.76
Cause of CKD diabetes (vs other/unknown)	2.23 (0.98, 5.09)	0.06
Cause of CKD autoimmune disease (vs other/unknown)	1.20 (0.45, 3.20)	0.71
Recipient history of coronary artery disease	0.99 (0.59, 1.65)	0.97
Recipient previous transfusions	1.16 (0.76, 1.77)	0.48
Recipient first transplantation	0.60 (0.32, 1.09)	0.61
Donor after cardiac arrest (*vs neurologically deceased*)	3.50 (2.09, 5.86)	<0.01
Donor age *(per 10 years higher)*	1.16 (1.00, 1.34)	0.02
Donor hypertension	1.55 (1.00, 2.37)	0.03
Donor smoking history	1.20 (0.81, 1.77)	0.38
Donor terminal serum creatinine *(per 10 umol/L higher)*	0.98 (0.92, 1.03)	0.53
Use of hypothermic perfusion pump	0.64 (0.39, 1.06)	0.11
Center 1	0.84 (0.52, 1.36)	0.26
Total ischemic time per op (*per 1 hour higher*)	1.04 (1.00, 1.09)	0.04
Transplant vintage *(per 1-year higher)*	0.98 (0.92, 1.04)	0.54

*CI, confidence interval; CKD, chronic kidney disease.

When DGF was defined as failure of serum creatinine to decrease by 10% or more in the first 3 days post transplant, or serum creatinine over 250 umol/l on post-transplant day 5 in the presence of scintigraphic evidence of acute tubular necrosis, 295 patients (53%) who were not exposed to ACEi/ARBs pre-transplant experienced DGF, while 148 (44%) of those who were exposed to ACEi/ARBs did (p=0.01). Results of univariable analyses can be found in **Supplementary Table 2**. The multivariable model showed a similar protective effect of ACEi/ARBs exposure at the time of transplant (OR 0.69, 95% CI 0.50, 0.94) (**Supplementary Table 3**).

To better account for confounding by donor characteristics, we then proceeded to an analysis of matched discordant pairs, *ie*, pairs of recipients who received a kidney from the same donor but where only one of the recipients was exposed to ACEi/ARB pre-transplant. We found 58 such recipient pairs (116 patients) in our cohort, amongst whom 19 episodes of DGF occurred. The magnitude of the association (effect size) was numerically similar (OR: 0.55, 95% CI 0.18-1.66) to that observed for the full cohort, although results were no longer significant due to the small number of patients and events.

We then explored whether there was a class effect in the association between RAAS blockers and DGF. The effect sizes for the exposure to ACEi alone (OR: 0.68, 95% CI 0.39, 1.20) and to ARB alone (OR: 0.62, 95% CI 0.40, 0.95) were of similar magnitude to that observed for the pooled analysis of exposure to ACEi/ARB combined (OR: 0.64, 95% CI 0.44, 0.93) (**Table 3A**). We could not detect a dose-response relationship, as both doses of ACEi and ARB higher and lower than the median of their respective distributions had effect sizes that were of similar magnitude (**Table 3B**). Information on specific dosage and agents of recipients exposed to ACEi and ARB pre-transplant is found in **Supplementary Table 4**.

**Table 3A:** Univariable associations between combined renin-angiotensin-aldosterone system (RAAS) blockers, ACE inhibitors (ACEi) alone, and angiotensin-2 receptor blockers (ARB) alone with delayed graft function.

Exposure to RAAS blockers	Exposure to ACEi alone	Exposure to ARB alone
OR: 0.64 95% CI: 0.44, 0.93	OR: 0.68 95% CI: 0.39, 1.20	OR: 0.62 95% CI: 0.40, 0.95

**Table 3B:** Univariable associations between low doses, high doses and all doses of angiotensin-2 receptor blockers (ARB) alone and ACE inhibitors (ACEi) alone with delayed graft function.

Exposure to ARBs irrespective of the dose	Exposure to ARBs Dose at or below median	Exposure to ARB Dose above median
OR: 0.62 95% CI: 0.40, 0.95	OR: 0.60 95% CI: 0.33, 1.08	OR: 0.66 95% CI 0.37, 1.18
Exposure to ACEi irrespective of the dose	Exposure to ACEi Dose at or below median	Exposure to ACEi Dose above median
OR: 0.68 95% CI: 0.39, 1.20	OR: 0.61 95% CI: 0.25, 1.45	OR: 0.77 95% CI: 0.35, 1.69

## Discussion

In this study, we demonstrated that recipient preoperative exposure to ACEi/ARBs is associated with a lower risk of DGF in the early postoperative period. Even if these medications were discontinued and not reinstated immediately after transplant, long-acting RAAS blockers could lower the risk of DGF through two possible mechanisms. First, ACEi/ARBs have shown protective effects against renal and cardiac IRI in animal models (17–19). For instance, in a murine model of coronary IRI, ACEi were shown to preserve endothelial relaxation, which in turn was associated with less arteriolar vasoconstriction and better microvascular perfusion (18). ACEi also reduced infarct size and the number of apoptotic cardiomyocytes around the necrotic area in a rat model of cardiac IRI (20). In a rat model of renal IRI, telmisartan pretreatment inhibited intrarenal depletion of antioxidants and lipid peroxidation while improving kidney function (21). Multiple other studies in rats confirmed that pre-treatment with ARBs or ACEi decreased the severity of renal IRI through antioxidant, anti-inflammatory and antiapoptotic mechanisms (6–10). The renoprotective effect of ACEi seems to be mostly present acutely at the time of reperfusion, where they reduce intrarenal and systemic angiotensin-II, attenuate intrarenal inflammation and apoptosis, while reducing tubular necrosis and epithelial sloughing (22). Second, ACEi/ARBs suppress the tubuloglomerular feedback, which accelerates recovery from AKI. Tissular hypoxia in the allograft during ischemia causes injury to the proximal tubules, leading to increased influx of sodium in the distal tubules, activation of macula densa and RAAS (23). Tubuloglomerular feedback protects the kidney from excessive loss of sodium but reduces glomerular filtration and aggravates AKI in the postoperative period. By inhibiting the RAAS, ACEi/ARBs suppress these phenomena.

In a retrospective cohort study that included 94 living donor kidney transplant recipients, the 40 recipients who used ACEI/ARBs during the perioperative period were less likely to have a reduction in serum creatinine of more than 75% by day 3 post transplant (24). In line with this finding, the investigators’ recommendation was to discontinue RAAS blockers 1 to 2 weeks before live donor kidney transplant. We hypothesize that the discrepancy between this study and ours stems from the difference in ischemic time between deceased and living donors. Given the short ischemic time associated with living donor transplantation, a potential beneficial effect of ACEi/ARBs on IRI may not be present. Furthermore, the polyuria associated with living kidney donation may make recipients more likely to experience volume depletion. In this condition, the hemodynamic effect of ACEi/ARBs may slow creatinine decrease post transplant.

In recipient of deceased donor kidneys, Heinze et al. reported that those using ACEi/ARBs pre- and post-transplant (treated as a time-dependent variable) had better allograft and patient survival 10 years after transplantation (25), which was also true in the subgroup of patients who had experienced DGF (26).

We found only one prior study that examined the same question as ours, *ie*, whether pre-transplant exposure to ACEi/ARBs was associated with the risk of developing DGF (27). However in contrast to our study, ACEi/ARBs were given both pre- and post-transplant. The investigators reported that in 260 deceased donor kidney transplant recipients, pre-, peri- and post-operative ACEi/ARBs use was associated with a faster recovery from DGF (27). The incidence of DGF was 20% in ACEi/ARBs users versus 25% in non-users. Although this difference was reported as not significant due to a small sample size, these numbers are in line with our findings of a lower risk of DGF in pre-transplant ACEi/ARBs users. In addition, our large sample size allowed us to adjust for multiple potential confounding factors, including the use of hypothermic pump. Taken together, the 2 studies suggest a beneficial role for pre-transplant ACEi/ARBs exposure to reduce the incidence of DGF. We show that even if long-acting ACEi/ARBs are not represcribed immediately after transplant for fear of hyperkalemia or AKI, their pre-transplant use while patients are wait-listed and prescription immediately prior to the surgical procedure could still prove useful in preventing DGF. If the absolute risk reduction in future clinical trials are similar to the one we observed, the number needed to treat to prevent one episode of DGF would be 17. As over 85% of patients with stage 5 CKD suffer from hypertension (28) and only 38% of transplant candidates in our cohort were exposed to ACEi/ARBs, the use of ACEi/ARBs as a first-line agent for blood pressure control in wait-listed patients is a strategy that deserves to be tested.

We performed separate analyses to explore whether exposure to ACEi and ARBs had different associations with DGF, but the differences observed were minimal and not clinically significant. Our results suggest that RAAS inhibitors-both ACEi and ARB- share properties that can protect the kidney from IRI, which is in line with experimental studies in animals. We could not detect any dose-response relationship either, which suggests that most doses that were used clinically are associated with a lower incidence of DGF. However, our study was not powered to detect whether the extremes of the dose range could have different impact on DGF.

Our study has strengths, such as a large sample size and the inclusion of multiple variables in the in the analysis to adjust for potential confounding. Our findings were also robust to variations in the definition of DGF. The generalizability of our study is however limited by the fact that it involves only 2 centers and includes a predominantly Caucasian population at low immunological risk. Furthermore, we had no access to the duration of ACEi/ARB use prior to transplantation as most patients were previously followed in other centers.

In conclusion, the present study suggests that pre-transplant use of ACEi/ARBs is associated with a decreased risk of DGF in early postoperative period. DGF is associated with a higher risk of long-term graft loss, longer hospital stay and higher costs associated with transplantation. We believe that randomized controlled trials of systematic use of ACEi/ARBs in patients who are waiting for a deceased donor kidney transplant are needed to evaluate whether this strategy can lower the occurrence of DGF, which could improve clinical outcomes for a large number of transplant patients.

## Data Availability

The original contributions presented in the study are included in the article/**Supplementary Materials**, further inquiries can be directed to the corresponding author/s.

## Supplementary material

Supplementary Table 1 Associations between recipient, donor, and procedure characteristics and DGF requiring dialysis in univariable analyses (n=897)

Supplementary Table 2 Associations between recipient, donor, and procedure characteristics and DGF with or without dialysis (extended definition*) in univariable analyses (n=897)

Supplementary Table 3 Associations between recipient, donor, and procedure characteristics and DGF with or without dialysis (extended definition*) in final multivariable analyses (n=897)

Supplementary Table 4 Specific agents and doses of ACE inhibitors and angiotensin-receptor blockers used pre-transplant
