# Supplementary material for: Exposure to renin-angiotensin system inhibitors before kidney transplantation is associated with a decreased risk of delayed graft function
Source: Front Immunol. 2024 Nov 22;15:1447638. doi: 10.3389/fimmu.2024.1447638 (PMC11621105; doi:10.3389/fimmu.2024.1447638)
Supplement: Supplementary Table 1 — Associations between recipient, donor, and procedure characteristics and DGF requiring dialysis in univariable analyses (n=897) [file DataSheet1.docx]

Supplementary Table 1. Associations between recipient, donor, and procedure characteristics and DGF requiring dialysis in univariable analyses (n=897)

| **Characteristics** | **Univariable**  **odds ratio (95% CI**^a^**)** | ***p-*value** |
| --- | --- | --- |
| Recipient ACEi/ARBs use at transplantation | 0.64 (0.44, 0.93) | 0.02 |
| Recipient age at transplant (*per 10 years higher*) | 1.00 (0.88, 1.15) | 0.97 |
| Recipient African American ethnicity *(vs all other ethnicities)* | 2.11 (1.29, 3.46) | 0.003 |
| Female recipient (*vs male*)  Recipient obesity(body mass index ≥30 kg/m^2^) | 1.00 (0.70, 1.43)  2.47 (1.71, 3.54) | 0.98  <0.0001 |
| Recipient diabetes | 1.40 (0.97, 2.02) | 0.08 |
| Recipient prior coronary artery disease | 1.38 (0.91, 2.11) | 0.13 |
| Recipient positive CMV serology | 1.50 (1.06, 2.13) | 0.02 |
| Recipient statin use at transplant | 1.05 (0.74, 1.48) | 0.79 |
| Recipient active smoking at transplantation *(vs never smoked)* | 0.73 (0.43, 1.24) | 0.25 |
| Recipient past history of smoking *(vs never smoked)* | 1.15 (0.81, 1.62) | 0.44 |
| Recipient with polycystic kidney diseases *(vs other or unknown CKD cause)* | 0.65 (0.32, 1.29) | 0.22 |
| Recipient with glomerular disease *(vs other or unknown CKD cause)* | 1.34 (0.80, 2.24) | 0.26 |
| Recipient with autoimmune kidney disease *(vs other or unknown CKD cause)* | 1.05 (0.40, 2.76) | 0.93 |
| Recipient with diabetic kidney diseases *(vs other or unknown CKD cause)* | 1.76 (1.00, 3.09) | 0.05 |
| Recipient with hypertensive kidney disease *(vs other or unknown CKD cause)* | 1.78 (0.93, 3.40) | 0.08 |
| First transplant (vs retransplants)  Recipient pregnancy | 0.63 (0.39, 1.03)  1.63 (1.01, 2.64) | 0.06  0.05 |
| Donor-recipient HLA mismatch 3-4 (*vs 0-2*) | 1.06 (0.64, 1.73) | 0.83 |
| Donor-recipient HLA mismatch 5-6 (*vs 0-2*) | 1.04 (0.61, 1.76) | 0.89 |
| Absence of pre-transplant transfusions | 0.69 (0.48, 1.01) | 0.06 |
| Recipient pre-transplant class 1 anti-HLA> 0% by flow cytometry | 1.18 (0.83, 1.68) | 0.37 |
| Recipient pre-transplant class 2 anti-HLA> 0% by flow cytometry | 1.14 (0.77, 1.70) | 0.52 |
| Thymoglobulin induction | 3.48 (2.44, 4.96) | <0.0001 |
| Donor female sex | 1.07 (0.75, 1.50) | 0.73 |
| Donor CMV serology | 0.83 (0.58, 1.19) | 0.30 |
| Donor after cardiac arrest (*vs neurologically deceased*) | 2.34 (1.59, 3.50) | <0.0001 |
| Donor age *(per 10 years higher)* | 1.17 (1.05, 1.30) | 0.01 |
| Donor hypertension | 1.62 (1.12, 2.34) | 0.01 |
| Donor diabetes | 0.99 (0.53, 1.85) | 0.98 |
| Donor smoking | 1.39 (0.96, 2.00) | 0.08 |
| Donor peripheral vascular disease | 1.33 (0.78, 2.25) | 0.30 |
| Donor terminal serum creatinine *(per 10 umol/L higher)* | 0.97 (0.92, 1.02) | 0.17 |
| Donor height (*per 10 cm higher*) | 0.95 (0.84, 1.07) | 0.38 |
| Use of hypothermic perfusion pump | 0.67 (0.37, 1.23) | 0.19 |
| Center 1 | 0.56 (0.40, 0.80) | 0.001 |
| Total ischemic time (*per 1 hour higher*) | 1.04 (1.01, 1.08) | 0.02 |
| Transplant vintage *(per 1 year higher)* | 0.99 (0.94, 1.04) | 0.76 |
|  |  |  |

^a^*CI*, confidence interval.

Supplementary Table 2. Associations between recipient, donor, and procedure characteristics and DGF with or without dialysis (extended definition*) in univariable analyses (n=897)

| **Characteristics** | **Univariable**  **odds ratio (95% CI*)** | ***p-*value** |
| --- | --- | --- |
| Recipient ACEi/ARBs use at transplantation | 0.70 (0.54, 0.92) | 0.01 |
| Recipient age at transplant (*per 10 years higher*) | 1.19 (1.07, 1.32) | 0.001 |
| Recipient African American ethnicity *(vs all other ethnicities)* | 1.39 (0.89, 2.17) | 0.15 |
| Female recipient (*vs male*)  Recipient obesity (body mass index ≥30 kg/m^2^) | 0.93 (0.71, 1.22)  1.58 (1.16, 2.14) | 0.58  0.004 |
| Recipient diabetes | 1.82 (1.35, 2.46) | <0.0001 |
| Recipient prior coronary artery disease | 1.95 (1.37, 2.78) | 0.0002 |
| Recipient positive CMV serology | 1.30 (1.00, 1.70) | 0.05 |
| Recipient statin use at transplant | 0.92 (0.71, 1.20) | 0.54 |
| Recipient active smoking at transplantation *(vs never smoked)* | 0.78 (0.54, 1.14) | 0.20 |
| Recipient past history of smoking *(vs never smoked)* | 1.28 (0.98, 1.67) | 0.07 |
| Recipient with polycystic kidney diseases *(vs other or unknown CKD cause)* | 1.01 (0.65, 1.59) | 0.95 |
| Recipient with glomerular disease *(vs other or unknown CKD cause)* | 1.08 (0.74, 1.58) | 0.69 |
| Recipient with autoimmune kidney disease *(vs other or unknown CKD cause)* | 1.05 (0.52, 2.11) | 0.89 |
| Recipient with diabetic kidney diseases *(vs other or unknown CKD cause)* | 2.08 (1.33, 3.25) | 0.001 |
| Recipient with hypertensive kidney disease *(vs other or unknown CKD cause)* | 1.25 (0.75, 2.09) | 0.39 |
| First transplant (vs retransplants)  Recipient past pregnancy | 0.82 (0.54, 1.23)  1.02 (0.76, 1.37) | 0.33  0.89 |
| Donor-recipient HLA mismatch 3-4 (*vs 0-2*) | 1.38 (0.95, 2.02) | 0.09 |
| Donor-recipient HLA mismatch 5-6 (*vs 0-2*) | 1.31 (0.87, 1.95) | 0.19 |
| Absence of pre-transplant transfusions | 0.72 (0.54, 0.85) | 0.02 |
| Recipient pre-transplant class 1 anti-HLA> 0% by flow cytometry | 1.20 (0.91, 1.59) | 0.19 |
| Recipient pre-transplant class 2 anti-HLA> 0% by flow cytometry | 1.17 (0.86, 1.60) | 0.33 |
| Thymoglobulin induction | 2.14 (1.59, 2.89) | <0.0001 |
| Donor female sex | 1.18 (0.90, 1.53) | 0.23 |
| Donor CMV serology | 1.01 (0.77, 1.33) | 0.93 |
| Donor after cardiac arrest (*vs neurologically deceased*) | 3.40 (2.33, 4.97) | <0.0001 |
| Donor age *(per 10 years higher)* | 1.30 (1.19, 1.42) | <0.0001 |
| Donor hypertension | 1.40 (1.04, 1.89) | 0.03 |
| Donor diabetes | 0.94 (0.58, 1.51) | 0.78 |
| Donor smoking | 1.32 (1.01, 1.74) | 0.04 |
| Donor peripheral vascular disease | 1.41 (0.91, 2.19) | 0.12 |
| Donor terminal serum creatinine *(per 10 umol/L higher)* | 1.07 (1.03, 1.11) | 0.0008 |
| Donor height (*per 10 cm higher*) | 0.81 (0.72, 0.91) | 0.0005 |
| Use of hypothermic perfusion pump | 0.47 (0.29, 0.75) | 0.002 |
| Center 1 | 0.46 (0.35, 0.60) | <0.0001 |
| Total ischemic time (*per 1 hour higher*) | 1.00 (0.98, 1.04) | 0.51 |
| Transplant vintage *(per 1 year higher)* | 1.04 (1.00, 1.08) | 0.04 |
|  |  |  |

^*^*CI*: confidence interval; DGF-extended definition: the need for dialysis in the first week post transplant, the failure of serum creatinine to decrease by 10% or more in the first 3 days post transplant, or serum creatinine over 250 umol/l on post-transplant day 5 in the presence of scintigraphic evidence of acute tubular necrosis.

Supplementary Table 3. Associations between recipient, donor, and procedure characteristics and DGF with or without dialysis (extended definition*) in final multivariable analyses (n=897)

| **Characteristics** |  |  | **Multivariable**  **odds ratio (95% CI*)** | ***p*-value** | |
| --- | --- | --- | --- | --- | --- |
| Recipient ACEi/ARBs use at transplantation |  |  | 0.69 (0.50, 0.94) | 0.02 | |
| Recipient age at transplant (*per 10 years higher*) |  |  | 0.91 (0.78, 1.05) | 0.20 | |
| Recipient African American ethnicity *(vs all other ethnicities)* |  |  | 1.37 (0.80, 2.32) | 0.25 | |
| Recipient obesity |  |  | 1.61 (1.13, 2.29) | <0.01 | |
| Recipient diabetes |  |  | 1.04 (0.65, 1.65) | 0.88 | |
| Recipient positive CMV serology |  |  | 1.45 (1.06, 1.99) | 0.02 | |
| Recipient active smoking at transplantation *(vs never smoked)* |  |  | 1.02 (0.65, 1.60) | 0.93 | |
| Recipient past history of smoking *(vs never smoked)* |  |  | 1.20 (0.85, 1.68) | 0.30 | |
| Cause of CKD* glomerular disease *(vs other/unknown)* |  |  | 0.98 (0.63, 1.51) | 0.91 | |
| Cause of CKD hypertension/vascular *(vs other/unknown)* |  |  | 0.67 (0.36, 1.26) | 0.21 | |
| Cause of CKD polycystic kidney disease *(vs other/unknown)* |  |  | 0.86 (0.52, 1.45) | 0.58 | |
| Cause of CKD diabetes *(vs other/unknown)* |  |  | 1.54 (0.81, 2.94) | 0.19 | |
| Cause of CKD autoimmune disease *(vs other/unknown)* |  |  | 0.70 (0.31, 1.55) | 0.37 |  |
| Recipient history of coronary artery disease |  |  | 1.30 (0.86, 1.95) | 0.21 |  |
| Recipient previous transfusions |  |  | 1.20 (0.88, 1.64) | 0.25 |  |
| Donor-recipient 3-4 HLA mismatch *(vs 0-2)* |  |  | 1.10 (0.71, 1.69) | 0.67 | |
| Donor-recipient 5-6 HLA mismatch *(vs 0-2)* |  |  | 1.08 (0.67, 1.74) | 0.74 | |
| Donor after cardiac arrest (*vs neurologically deceased*) |  |  | 4.77 (3.10, 7.35) | <0.01 | |
| Donor age *(per 10 years higher)* |  |  | 1.34 (1.19, 1.51) | <0.01 | |
| Donor hypertension |  |  | 0.99 (0.69, 1.42) | 0.94 | |
| Donor peripheral vascular disease |  |  | 0.87 (0.53, 1.42) | 0.57 | |
| Donor smoking |  |  | 1.14 (0.84, 1.56) | 0.40 | |
| Donor terminal serum creatinine *(per 10 umol/L higher)* |  |  | 1.15 (1.09, 1.21) | <0.01 | |
| Donor height *(per 10 cm higher)* |  |  | 0.73 (0.63, 0.85) | <0.01 | |
| Use of hypothermic perfusion pump |  |  | 0.58 (0.39, 0.86) | <0.01 | |
| Center 1 |  |  | 0.67 (0.47, 0.96) | 0.03 | |
| Transplant vintage *(per 1-year higher)* |  |  | 1.03 (0.98, 1.08) | 0.23 | |
|  |  |  |  |  | |

* CI, confidence interval; CKD chronic kidney disease; DGF-extended definition: the need for dialysis in the first week post transplant, the failure of serum creatinine to decrease by 10% or more in the first 3 days post transplant, or serum creatinine over 250 umol/l on post-transplant day 5 in the presence of scintigraphic evidence of acute tubular necrosis.

Supplementary Table 4. Specific agents and doses of ACE inhibitors and angiotensin-receptor blockers used pre-transplant

| ACE inhibitors | Number of users* | Median daily dose  and range |
| --- | --- | --- |
| Perindopril  Ramipril  Enalapril  Fosinopril  Trandolapril  Lisinopril  Quinapril | 21  34  11  18  10  7  1 | 4 mg (2-12 mg)  10 mg (1.25-12.5 mg)  10 mg (2.5-20 mg)  20 mg (5-40 mg)  4 mg (1-8 mg)  10 mg (2.5-40 mg)  10 mg |
| Angiotensin-receptor blockers |  |  |
| Irbesartan  Candesartan  Valsartan  Losartan  Telmisartan  Olmesartan | 101  56  37  11  8  1 | 150 mg (37.5-300 mg)  16 mg (2-32 mg)  160 mg (40-320 mg)  100 mg (25-100 mg)  60 mg (40-80 mg)  20 mg |

*The total number is 316 instead of 337, since although exposure to ACEi/ARBs was confirmed, information on specific agent/dose use could not be retrieved from 21 files.
